# Synthesis, conjugating capacity and biocompatibility evaluation of a novel amphiphilic polynorbornene

**DOI:** 10.1080/15685551.2020.1812832

**Published:** 2020-08-30

**Authors:** Hengxi He, Bin Song, Guirong Qiu, Weixiang Wang, Haibin Gu

**Affiliations:** a Key Laboratory of Leather Chemistry and Engineering of Ministry of Education, Sichuan University, Chengdu, China; b College of Food and Bioengineering, Xihua University, Chengdu, China

**Keywords:** Polynorbornene, ring-opening metathesis polymerization, polymeric carrier, conjugate, pH-responsive

## Abstract

Polynorbornenes, prepared by the ‘living’ and ‘controlled’ ring-opening metathesis polymerization (ROMP) method, have emerged as a stimuli-sensitive new class of polymer carriers. Herein, we reported a novel amphiphilic diblock polynorbornene, **PNCHO-*b*-PNTEG**, containing active benzaldehyde units, which exhibited good conjugating capacity to amino-containing molecules (e.g., doxorubicin (DOX)) via the pH-sensitive Schiff base linkage. The copolymer and its conjugate with DOX, **DOX-PNCHO-*b*-PNTEG**, were adequately analyzed by various techniques including ^1^H NMR, ^13^C NMR, gel permeation chromatography, etc. Especially, the formed conjugate of **DOX-PNCHO-*b*-PNTEG** could self-assemble into near-spherical micelles with the diameter of 81 ± 10 nm, and exhibit acid-triggered DOX release behavior, and the release rate could be adjusted by changing the environmental pH value. The excellent biological safety of **PNCHO-*b*-PNTEG** was further demonstrated by the results from both *in vitro* toxicity evaluation to murine fibroblast cells (L-929 cells) and *in vivo* evaluation of acute developmental toxicity and cell death in zebrafish embryos. Hence, the present polynorbornene-based **PNCHO-*b*-PNTEG** possesses great potential application as a biocompatible polymeric carrier and could be employed to fabricate various pH-sensitive conjugates.

## Graphical abstract

**Figure:**
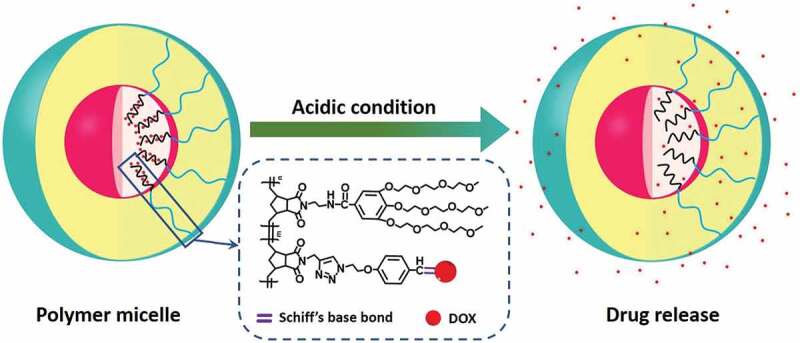

## Introduction

Amphiphilic polymers were widely designed and investigated by researchers in the past many years owing to their extensive smart applications as carriers in materials, catalysis, and medicine. Various stimuli, including temperature [1], ultrasound [2], light [3], redox [4], and pH [5], could be integrated into the polymeric carriers formed by amphiphilic polymers, which is, in particular, beneficial for the construction of smart drug delivery systems (DDSs). Thereinto, the pH-sensitive conjugates between amphiphilic polymers and drug molecules have emerged as a shining platform attracting intensive interest of researchers [6–8]. Normally, the amphiphilic polymers exhibited the conjugating capacity to drug molecules through the acid-sensitive chemical linkages such as hydrazone [9], Schiff base [10], oxime [11], acetal [12], ketal [13], amide [14], ether [15], orthoester bonds [16], etc. Especially, owing to the easy synthesis and desirable pH-sensitivity, the Schiff base bond was often adopted to graft drugs to various polymeric carriers for the construction of the pH-sensitive conjugates [17,18].

The selection of an appropriate polymeric skeleton is also crucial for the fabrication, property, and application of polymeric carriers. Although many linear polymers, such as polyethylene (PE) [9], polyethylene glycol (PEG) [17], and polysaccharide [8], were typically used by researchers as polymeric carriers, polynorbornenes, synthesized by the ‘living’ and ‘controlled’ ring-opening metathesis polymerization (ROMP) technique [19,20], have been established recently as a new class of polymer backbone for the preparation of polymeric carriers [21–24]. For example, Zou et al. [25] reported the pH-sensitive brush conjugates synthesized by the ROMP of two norbornene-based macromonomers containing paclitaxel (PTX) and PEG, respectively. The PTX molecules were covalently attached to polynorbornene backbones as pendent groups *via* the acid-sensitive cycloacetal linkages, and thus could be selective and rapid released under acidic conditions. Using ROMP, Mukherjee et al. [26] synthesized a new polynorbornene-based triblock copolymer that could covalently combine anticancer drug of doxorubicin (DOX), MRI agent of cobalt carbonyl complex and biotin in the side chain. The obtained conjugate with high content of DOX showed acid-triggered enhanced release behavior due to the acid-sensitive hydrazone-based linkage between DOX moieties and polynorbornene backbones, and exhibited excellent MRI contrast eﬀects and transverse relaxation properties, too [26]. Unfortunately, most of the known reports just confirmed the feasibility of the synthesized polynorbornenes as polymeric carriers for the construction of conjugates, but their biocompatibility has seldom been evaluated through *in vitro* and *in vivo* experiments. Moreover, most of the reported polynorbornene-based amphiphilic polymers exhibited moderate or low reaction reactivity to graft various kinds of molecules.

**Scheme 1.**
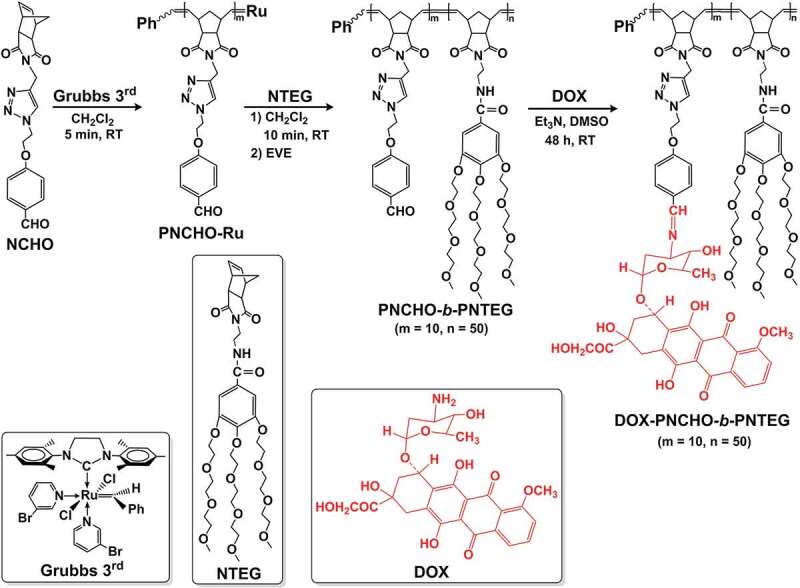
Synthesis routes of **PNCHO-*b*-PNTEG** and **DOX-PNCHO-*b*-PNTEG**

In this study, we design a new polynorbornene-based amphiphilic block copolymer **PNCHO-*b*-PNTEG** (Scheme 1) with high reaction reactivity to graft various kinds of amino-containing compounds. This amphiphilic block copolymer contains hydrophobic reactive benzaldehyde units and hydrophilic dendritic triethylene glycol (TEG) moieties in the side chain. This copolymer was firstly and rapidly controlled synthesized in a short time (15 min in all) with 100% conversion via the ROMP method with the help of the Grubbs’ 3rd generation catalyst (**Grubbs 3^rd^**, Scheme 1). Then, the formed **PNCHO-*b*-PNTEG** exhibited 100% of grafting rate to amino-containing molecules (e.g., DOX) via the pH-sensitive Schiff base linkage. Besides synthesis and characterization, the self-assembly behavior and *in vitro* DOX release of the obtained conjugate **DOX-PNCHO-*b*-PNTEG** (Scheme 1) were also addressed in this paper. Finally, the *in vitro* and *in vivo* biotoxicity evaluations of this polymeric carrier **PNCHO-*b*-PNTEG** were conducted by using murine fibroblast cells (L-929 cell) and model organism of zebrafish embryos, respectively, and the excellent biological safety of this new polymeric carrier was well confirmed.

## Experimental

### Materials

Monomer **NCHO** [27], monomer **NTEG** [28], and **Grubbs 3^rd^** catalyst [29] were synthesized following the reported methods in the literature. The details for other materials and instruments can be found in the Supporting Information (SI).

### Synthesis of PNCHO-b-PNTEG

Monomers **NCHO** (20.0 mg, 0.0529 mmol, 10 equiv) and **NTEG** (210.7 mg, 0.2643 mmol, 50 equiv) were dissolved in 0.5 mL and 2.0 mL, respectively, of dry dichloromethane (CH_2_Cl_2_). The freshly prepared **Grubbs 3^rd^** catalyst (4.69 mg, 0.00528 mol, 1 equiv) was added into a dry glass bottle and dissolved by 0.2 mL of dry CH_2_Cl_2_. The solution of **NCHO** was firstly added rapidly into the glass bottle, and the obtained mixture was then dramatically agitated at R.T. (25°C or so) for 5 min under N_2_ protection. Next, the solution of **NTEG** was injected rapidly into the glass bottle, and after the addition, the resulted mixture was vigorously agitated for 10 min at R.T. under N_2_ protection. Finally, 0.5 mL of ethyl vinyl ether (EVE) was injected into the reaction container to quench the polymerization. The final reaction mixture was distilled via a rotary evaporator to remove the solvent. The residue was then dissolved in a very small amount of dry CH_2_Cl_2_, and diethyl ether was added to separate the product as precipitate. The above procedure was repeated twice, and the precipitate was collected and dried under vacuum at R.T. to constant weight. The targeted **PNCHO-*b*-PNTEG** was obtained as dark green solid with the yield of 95%. ^1^H NMR (400 MHz, (CD_3_)_2_SO, 25°C, TMS) δ_ppm_: 9.82 (s, 1H, CHO), 8.43 (broad, 5 H, NHCO), 8.02 (broad, 1H, C = CH of triazole), 7.81 (broad, 2 H, ph of CHO block), 7.41–7.29 (m, end-ph), 7.08 (s, 12 H, ph of CHO and TEG blocks), 5.50 and 5.45 (broad, 12 H, CH = CH of polynorbornene), 4.72 (broad, 2 H, NCH_2_-triazole), 4.56 and 4.47 (broad, 4 H, NCH_2_CH_2_O), 4.10 and 4.04 (ds, 30 H, 3 × ph-OC***H_2_***), 3.73–3.37 (m, CON-C***H***_2_C***H***_2_-NHCO and 3 × OC***H***_2_C***H***_2_OC***H***_2_C***H***_2_O of the TEG block), 3.21 and 3.20 (ds, 45 H, 3 × OCH_3_), 2.99 (broad, = CH-C***H***), 2.61 (broad, CHCON), 1.91 and 1.42 (broad, CH_2_ of cyclopentane). ^13^C NMR (100 MHz, CDCl_3_, 25°C, TMS) δ_ppm_: 190.9 (CHO), 178.6 (NC = O), 167.2 (NHC = O), 163.5, 133.5, 129.2, 114.9 (ph of CHO block), 152.3, 140.8, 130.4, 106.6 (ph of TEG block), 140.8 and 124.0 (triazole of CHO block), 132.0 and 130.4 (C = C of polynorbornene), 72.3, 71.9, 70.7, 70.5, 70.4, 59.6, 68.8 and 68.7 (OCH_2_), 65.0 (triazole-CH_2_***C***H_2_ of CHO block), 58.9 (-OCH_3_), 52.4 (triazole-***C***H_2_ of CHO block), 50.9 (***C***HCON), 45.5 (=CHCH***C***H_2_), 42.9 (=CH-***C***H), 41.0 (N***C***H_2_CH_2_NHCO), 38.1 (NCH_2_***C***H_2_NHCO), 29.6 (CON-***C***H_2_-triazole of CHO block).

### Synthesis of DOX-PNCHO-b-PNTEG

Doxorubicin hydrochloride (DOX·HCl, 37.5 mg, 0.0646 mmol, 2 equiv) was dissolved in 1 mL of DMSO in a glass vial, and trimethylamine (Et_3_N, 32.6 mg, 0.323 mmol, 10 equiv) was then added and stirred at R.T. in dark for 2 h to neutralize HCl. The obtained mixture was injected into the DMSO solution (1.5 mL) of **PNCHO-*b*-PNTEG** (141.5 mg, 0.0323 mmol of CHO, 1 equiv), agitated vigorously at R.T. in dark for 48 h, transferred to a dialysis bag with molecular weight cut off (MWCO) of 3500 Da, and dialyzed against deionized water for 72 h to remove the residual DMSO, Et_3_N, excessive DOX and the formed salts. During this period, the dialysate was replaced by fresh deionized water every 12 h. The final product in the dialysis bag was then lyophilized for 72 h to get the dried conjugate of **DOX-PNCHO-*b*-PNTEG**. Similar procedures were adopted for the grafting of other model molecules including *O*-benzylhydroxylamine, 1-hexadecanamine, tryptophan, and benzocaine.

### Preparation of micelles of PNCHO-b-PNTEG and DOX-PNCHO-b-PNTEG

5 mg of **PNCHO-*b*-PNTEG** was dissolved at R.T. in 5 mL of DMSO, followed by the dropwise addition of 5 mL deionized water under vigorously stirring condition. The obtained mixture was then dramatically agitated at R.T. for 2 h, then transferred into a dialysis bag (MWCO = 3500 Da), and dialyzed against deionized water at R.T. for 72 h to remove the residual DMSO. The micelles of **PNCHO-*b*-PNTEG** were finally obtained with a concentration of 0.5 mg/mL. A similar procedure was adopted to fabricate micelles of **DOX-PNCHO-*b*-PNTEG** with a concentration of 0.5 mg/mL.

### pH-responsive release of DOX from the micelles of DOX-PNCHO-b-PNTEG

10 mL of the micelles of **DOX-PNCHO-*b*-PNTEG** was added in a dialysis bag (MWCO = 3500 Da) and immersed in 100 mL of PBS buffer solutions with different pH values (4.0, 6.0, and 7.4, respectively) condition for pH-stimuli release. All the experiments were conducted at 37°C to mimic the internal ambient temperature of human body. At different time intervals, 2 mL of dialysate was taken out for the UV-vis detection at the wavelength 485 nm, and the dialysate was then added back to the dialysis bag after the UV detection for continued dialysis. The accumulation release amount of DOX was calculated using the DOX standard curve.

### Cell toxicity evaluation of PNCHO-b-PNTEG

Murine fibroblast cells (L-929 cells) were used for in vitro cell toxicity evaluation and the concentration range of **PNCHO-*b*-PNTEG** micelles used was 8.0–0.0625 mg/mL. All the micellar solutions were sterilized at 121°C for 20 min. A total of 100 μL of L929 cell suspension was added in a 96-well plate, and the plate was then placed in an incubator at 37°C for a 24 h pre-incubation under a humidified atmosphere containing 5% CO_2_. After that, 10 μL of the micellar solution was injected into the wells contain L929 cells and the mixture was further incubated for 24 h, 48 h, and 72 h, respectively, under the same conditions. Three sets of parallel experiments were conducted for each sample. Then, 10 μL of CCK 8 reagent was added to each well of the plate, and the plate was incubated at 37°C for 1.5 h. Next, a microplate reader (BIO-RAD550) was adopted to measure the absorbance of the culture medium at the wavelength of 450 nm. The absorbance values of Dulbecco’s Modified Eagle’s Medium (DMEM) with cells and without cells were set as positive and negative controls, respectively, and the cell relative proliferation rate (RPR) was determined using the following formula [28].

$$RPR\left(\%\right)=\frac{OD_{t}-OD_{n}}{OD_{p}-OD_{n}}\times 100\%$$

where *OD*_t_, *OD*_p_, and *OD*_n_ are the absorbance values of the test group, positive control, and negative control groups, respectively.

### Biotoxicity evaluation of PNCHO-b-PNTEG to zebrafish

The biotoxicity of micellar solutions of **PNCHO-*b*-PNTEG** to zebrafish embryos was tested according to the method reported in the literature [28]. All the animal procedures were conducted in terms of the Guidelines for Care and Use of Laboratory Animals of Xihua University, and all the experiments were permitted by the Animal Ethics Committee of Xihua University. The tested micellar concentrations were 8.0, 4.0, and 0.5 mg/mL, respectively. The formulas of the embryo culture medium (Holt buffer) contained 3.5 g of NaCl, 0.05 g of KCl, 0.025 g of NaHCO_3_, and 0.1 g of CaCl_2_ per liter. All the micellar solutions and the embryo culture medium were sterilized at 121°C for 20 min. Zebrafish embryos were collected from spawning zebrafish at 0.5–1 hpf (hour post fertilization) and placed in the fresh and sterilized embryo culture. Healthy embryos were checked and selected using a stereomicroscope (SZX10, Olympus, Japan) and transferred into a 6-well cell culture plate with 5 mL of Holt buffer (20 embryos per well). At 6 hpf, embryos were treated with different concentrations of micellar solutions of **PNCHO-*b*-PNTEG**. Three groups of experiments were carried out in parallel for each experimental concentration. The embryo mortality, malformation rate, and hatching rate were observed at 24, 48, and 72 hpf, respectively, to evaluate the developmental toxicity of **PNCHO-*b*-PNTEG** to zebrafish embryos.

### Detection of apoptosis of zebrafish

Acridine orange (AO) solution (2 mg/L) was prepared by using the embryo culture medium as solvent. The hatched larvae at 96 hpf were stained with AO solution at 28.5°C for 30 min in the dark and then washed with Holt buffer for 3 times (5 min per time). The obtained zebrafish larvae were anesthetized for 5 min using 0.08% of 2-phenoxyethanol, and then placed on the glass slide for observation using fluorescence microscope at 450 nm or blue light (B), and each fish was photographed at the five different angles. The enhanced green color will be observed at the site of aggravation of apoptosis, while weakened green color will be found at the inhibitory site. The fluorescence intensity of images was quantified using ImageJ software (National Institutes of Health, NIH). Results are expressed as the percent change in the control medium. All the experiments were conducted in triplicate.

### Statistical analysis

Statistical analyses were conducted by one-way ANOVA using SPSS 26.0. Multiple comparisons versus control were completed using Dunn’s method, with significance at the p < 0.05 level. All the experiments were carried out in triplicate and all data were expressed as means ± S.D.

## Results and discussion

### Synthesis of PNCHO-b-PNTEG

The monomers **NCHO** and **NTEG** were prepared by using the methods in our previous reports [24,28]. And, results of their ROMP kinetic studies indicated that it took only 5 and 10 min, respectively, to achieve the 100% conversion for the ROMP reactions of **NCHO** and **NTEG**. Thus, their rapid ROMP rates are expected to ease the difficulty level and shorten the reaction time for the synthesis of the targeted diblock copolymer **PNCHO-*b*-PNTEG**. Concretely, as shown in Scheme 1, the monomer **NCHO** was first subjected to ROMP polymerization at R.T. in dry CH_2_Cl_2_. A homopolymer with ruthenium-end, **PNCHO-Ru**, was obtained and subsequently applied as a macroinitiator to further catalyze the ROMP reaction of hydrophilic **NTEG** monomer. The adopted feed molar ratio of **NCHO, NTEG**, and **Grubbs 3^rd^** catalyst was 10:50:1. The ROMP of **NCHO** was achieved within 5 min in dry CH_2_Cl_2_ with a 100% conversion rate. The kinetic study for the second **NTEG** block was conducted *via* the *in situ* ^1^H NMR analysis method. As shown in Figure S14, only 10 min was needed to conduct the RMOP of **NTEG** with 100% monomer conversion to form the second block of **PNCHO-*b*-PNTEG**.

**Figure 1.**
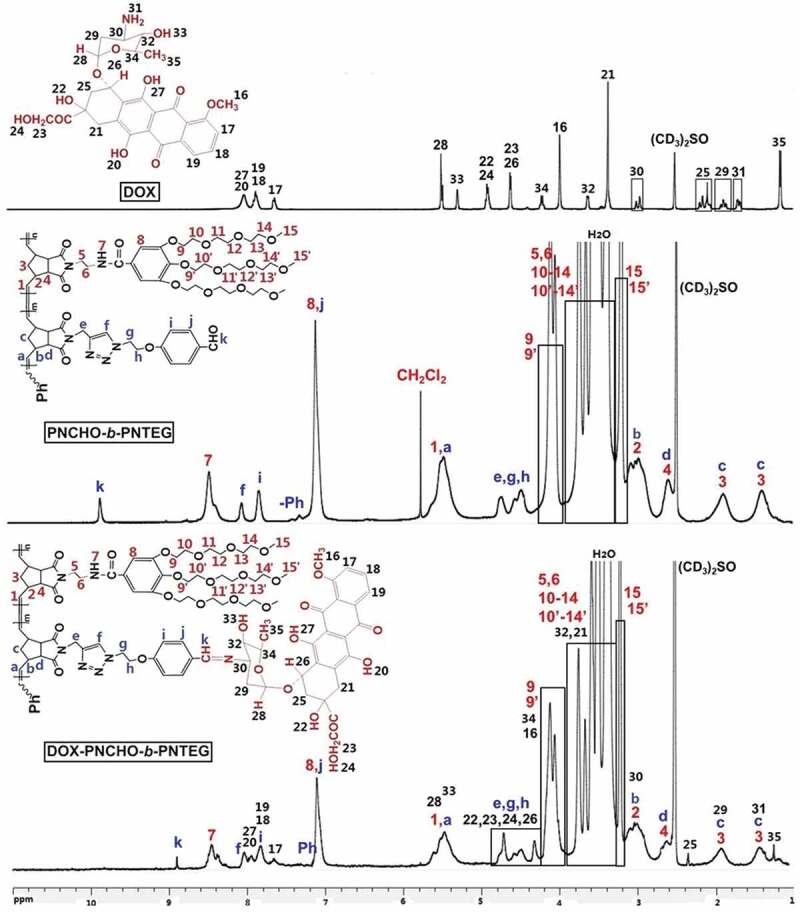
^1^H NMR spectra of DOX, **PNCHO-*b*-PNTEG** and **DOX-PNCHO-*b*-PNTEG** in (CD_3_)_2_SO

**Figure 2.**
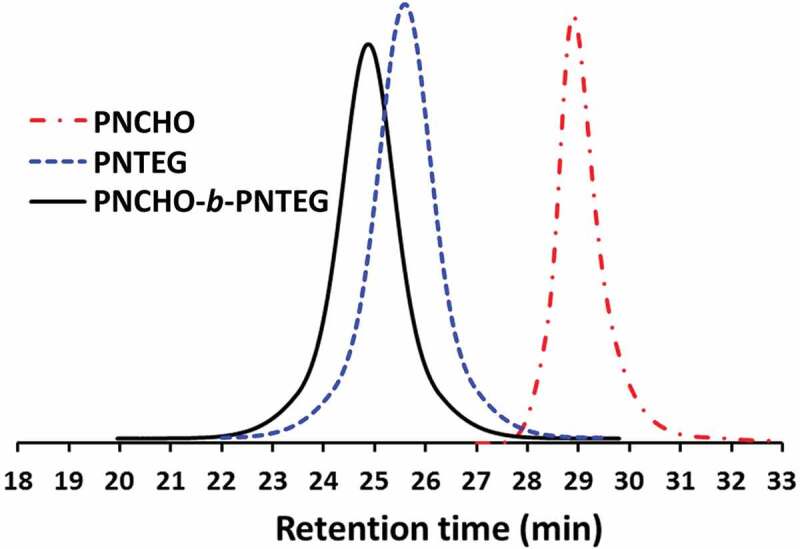
GPC curves of **PNCHO, PNTEG**, and **PNCHO-*b*-PNTEG** in DMF

^1^H NMR spectrum of **PNCHO-*b*-PNTEG** is provided in Figure 1, and the assignments for all the peaks were clearly marked. This analysis powerfully demonstrated the formation and structure of the targeted diblock copolymer. Its ^13^C NMR (Figure S11), IR (Figure S12) and UV-vis (Figure S13) spectra were also provided in the SI, and the corresponding analysis results further indicated its successful synthesis. The molecular weight (MW) of **PNCHO-*b*-PNTEG** was determined using the ^1^H NMR end-group analysis (Table S2 and S5) and GPC methods, respectively, and the results were compared with those of **PNCHO** and **PNTEG**, as shown in Table 1. Notably, all the theoretical M_1_ values are larger than the M_n_ values from GPC measurements, owing to the large structural difference between the PS standards and these polymers [27]. According to the end-group analysis, the obtained results of polymer degrees for the first and the second blocks are 10 ± 0.5 and 50 ± 1, respectively. As shown in Figure 2, the GPC curve of **PNCHO-*b*-PNTEG** is compared with those of **PNCHO** and **PNTEG** whose feed molar ratios are 10:1 and 50:1, respectively. An obvious forward shift retention time is found in comparison to the GPC curves of **PNCHO** and **PNTEG**, which undisputedly proves the increase of MW of **PNCHO-*b*-PNTEG** as a result of its successful synthesis. The detailed description demonstrating the synthesis, structure, and MW of **PNCHO-*b*-PNTEG** can be found in the 4.4 section of the SI.

**Table 1.** MW data of **PNCHO, PNTEG**, and **PNCHO-*b*-PNTEG.**

Polymer	PNCHO	PNTEG	PNCHO-b-PNTEG	
[M].:[C].^a^	10: 1	50: 1	10: 50: 1	
Conv^b^(%)	>99	>99	>99	>99
n_p1_^c^	10	50	10	50
n_p2_^d^	10 ± 0.5	–	10 ± 0.5	50 ± 1
M_1_^e^	4 028	39 949	43 873	
M_n_^f^	18 414	54 615	68 323	
PDI^g^	1.10	1.14	1.21	

^a^[M].:[C].: feed molar ratio of monomer to **Grubbs 3^rd^**. ^b^ Monomer conversion calculated *via* ^1^H NMR spectroscopy. ^c^ Polymerization degree obtained from ^1^H NMR data using the monomer conversion. ^d^ Polymerization degree calculated using the ^1^H NMR end-group analysis method. ^e^ Theoretical MW calculated by the conversion of monomer. ^f^ MW determined by GPC with PS (polystyrene) as the standard. ^g^ PDI calculated from the GPC curve.

### Synthesis of DOX-PNCHO-b-PNTEG

**Figure 3.**
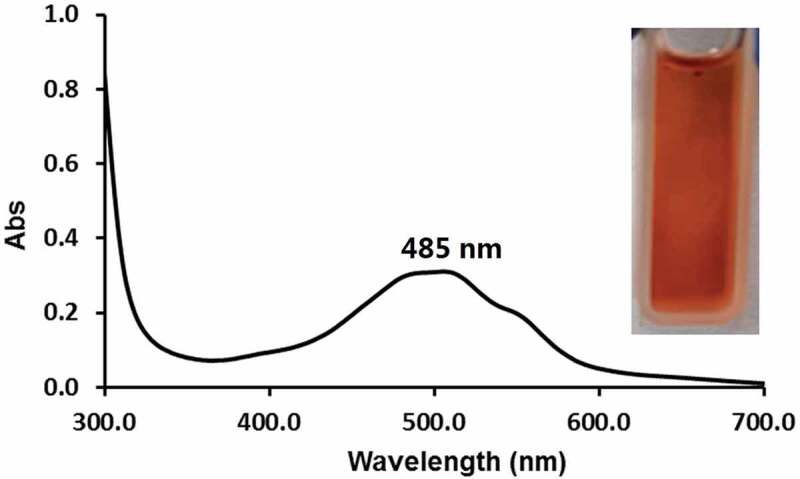
UV-vis spectrum of **DOX-PNCHO-*b*-PNTEG** in H_2_O and its picture

The reactive aldehyde groups of **PNCHO-*b*-PNTEG** were designed to graft amino-containing molecules by Schiff base bond (CH = N). To confirm its conjugating capacity, DOX was employed to react with **PNCHO-*b*-PNTEG** to prepare the conjugate of **DOX-PNCHO-*b*-PNTEG**. The grafting reaction was conducted at R.T. in DMSO with the presence of Et_3_N, and the obtained product was purified by the typical dialysis method. Then, the ^1^H NMR spectrum of the purified product was recorded. Figure 1 provides the comparison of ^1^H NMR spectra of DOX, **PNCHO-*b*-PNTEG**, and **DOX-PNCHO-*b*-PNTEG** in (CD_3_)_2_SO. As expected, after the grafting reaction, the peak of aldehyde proton at 9.82 ppm completely disappeared, and a new peak assigned to the CH = N proton was found at 8.90 ppm. These results demonstrate the successful synthesis of the aimed conjugate **DOX-PNCHO-*b*-PNTEG** by the Schiff base reaction between the CHO groups of the first block with the amino groups of DOX, and the grafting rate is 100%. Using the theoretical polymerization degrees of 10 and 50, respectively, for the first and second blocks of **PNCHO-*b*-PNTEG** (Table 1), the theoretical MW of **DOX-PNCHO-*b*-PNTEG** could be calculated to be 49,129 Da, and thus the DOX-loading content in this conjugate was determined to be 11.06% (wt). Furthermore, as shown in Figure 1, the other peaks in the ^1^H NMR spectrum of **DOX-PNCHO-*b*-PNTEG** were well assigned and match its expected structure, which further confirms the structural integrity of DOX and **PNCHO-*b*-PNTEG**. In the UV-vis spectrum of **DOX-PNCHO-*b*-PNTEG** (Figure 3), as expected, three peaks at 485, 510, and 540 nm are observed, which could be attributed to the characteristic absorption peaks of DOX. The appearance of these peaks confirms the successful grafting of DOX to **PNCHO-*b*-PNTEG**. Notably, unlike the case in Figure S20A (the UV-vis spectrum of free DOX), the intensity of peak at 510 nm is lightly stronger than that at 485 nm, which is attributed to the electron environment change of DOX resulted from the formation of conjugated structure of – N = CH-ph. Thus, the maximum absorption peak is the peak at 510 nm, not the peak at 485 nm. And it is worth to say the color of the solution changed from the original colorless to dark red, after the grafting reaction. The IR spectrum of **DOX-PNCHO-*b*-PNTEG** (Figure S15) also gives helpful information indicating the successful reaction between DOX and **PNCHO-*b*-PNTEG**. For example, the vibration peak of the CHO group at 1700 cm^−1^ in the structure of **PNCHO-*b*-PNTEG** disappeared, while a new peak corresponding to the stretching vibration of the CH = N bond appeared at 1600 cm^−1^. All these results unarguably confirm the successful conjugation of DOX to **PNCHO-*b*-PNTEG** via the Schiff base reaction and the expected structure of **DOX-PNCHO-*b*-PNTEG**.

The reactivity of aldehyde groups of **PNCHO-*b*-PNTEG** was further tested by using other amino-containing model compounds including *O*-benzylhydroxylamine, 1-hexadecaneamine, tryptophan, and benzocaine, and the partial ^1^H NMR spectra of the corresponding PDCs were shown in Figure S16. As expected, no peak of aldehyde proton was observed in all the ^1^H NMR spectra, while new CH = N proton peaks were found for all the conjugates. These results indicate the 100% grafting rate for all the tested model compounds. In short, the **PNCHO-*b*-PNTEG** exhibits high reaction reactivity to graft various kinds of amino-containing compounds, which is anticipated to have great potential as a new carrier system to fabricate various conjugates used for drug delivery.

### Self-assembly of PNCHO-b-PNTEG and DOX-PNCHO-b-PNTEG

It is well known that, owing to the presence of the hydrophilic and hydrophobic segments, amphiphilic block copolymers can self-assemble into fascinating nanostructures with various morphologies such as spheres, cylinders, vesicles, lamellae, and micelles [30,31]. In the present study, **PNCHO-*b*-PNTEG**, containing hydrophobic CHO block and hydrophilic TEG block, is expected to self-assemble into nanostructures in water. And after the grafting reaction with DOX, the resulting **DOX-PNCHO-*b*-PNTEG** is anticipated to keep the amphiphilicity due to the hydrophobic nature of DOX [32], and thus also has the potential to self-assemble into nanostructures in aqueous solution. To confirm this point, herein, the self-assembly behaviors of both **PNCHO-*b*-PNTEG** and **DOX-PNCHO-*b*-PNTEG** were investigated and compared.

The critical micelle concentrations (CMCs) of **PNCHO-*b*-PNTEG** and **DOX-PNCHO-*b*-PNTEG** were firstly measured using the typical pyrene fluorescence probe technique [30], and the details were described in Figure S17 and S19. The obtained CMC values are 0.126 mg/mL for **PNCHO-*b*-PNTEG** and 0.05 mg/mL for **DOX-PNCHO-*b*-PNTEG**, respectively. As expected, **DOX-PNCHO-*b*-PNTEG** has a lower CMC than **PNCHO-*b*-PNTEG**, which is attributed to the increased hydrophobicity and size of the CHO block after the conjugation of the hydrophobic DOX molecular. According to these CMC results, a concentration (0.5 mg/mL) above the corresponding CMC values was used for the following self-assembly experiments. The dialysis method [30,33] was applied to fabricate micellar aggregates of **PNCHO-*b*-PNTEG** and **DOX-PNCHO-*b*-PNTEG**. Both copolymers were dissolved in DMSO, followed by the dropwise addition of deionized water, vigorous stirring of 2 h and then dialysis against deionized water for 72 h. The Tyndall eﬀect (Figure S18) was clearly observed in the obtained aqueous solutions, which indicates the formation of aggregates through the self-assembly of **PNCHO-*b*-PNTEG**. The size and morphology of the obtained samples were characterized by SEM and DLS.

**Figure 4.**
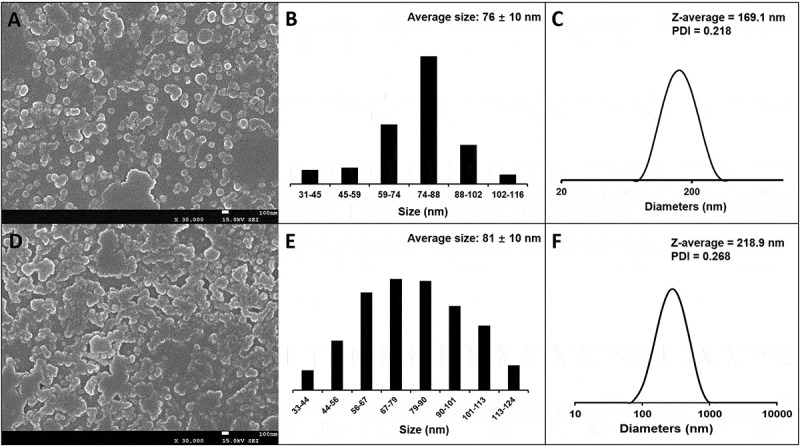
SEM images of micelles of **PNCHO-*b*-PNTEG** (a) and **DOX-PNCHO-*b*-PNTEG** (d). Size distribution by the SEM image of micelles of **PNCHO-*b*-PNTEG** (b) and **DOX-PNCHO-*b*-PNTEG** (e). DLS curve of micelles of **PNCHO-*b*-PNTEG** (c) and **DOX-PNCHO-*b*-PNTEG** (f)

The near-spherical micelles are observed by the SEM image of **PNCHO-*b*-PNTEG** (Figure 4(a)), and the corresponding statistical size analysis provided the average particle diameter of 76 ± 10 nm (Figure 4(b)). In a typical micelle, the aggregation of the hydrophobic CHO block led to the formation of dark core, while the self-assembly of hydrophilic TEG block resulted in the formation of the bright periphery [24]. Moreover, a hydrodynamic diameter of 169 nm with PDI (polydispersity index) of 0.218 is provided by the DLS curve (Figure 4(c)) of the micelles of **PNCHO-*b*-PNTEG**. Obviously, the micellar particle size from DLS is typically larger than the value obtained by SEM. This size difference could be caused by the different measurement conditions of DLS (wet micelles) and SEM (dried micelles) [24,28,30] and the slight aggregation of these micelles in aqueous solution. In short, the above results confirm that **PNCHO-*b*-PNTEG** could self-assemble into micelles on the nanoscale in aqueous solution.

The near-spherical morphology of **DOX-PNCHO-*b*-PNTEG** micelles also evidenced by the SEM image (Figure 4(d)), and the size distribution is found to be 81 ± 10 nm (Figure 4(e)). The hydrodynamic diameter of micelles of **DOX-PNCHO-*b*-PNTEG** is 219 nm with PDI (polydispersity index) of 0.268 (Figure 4(f)), and this value is dramatically larger than that reflected by SEM, which is primarily resulted from the slight aggregation in this micellar system as shown in Figure 4(d). Compared to the micelles of **PNCHO-*b*-PNTEG**, the micelles of its conjugate exhibit obviously bigger particle size as indicated by both SEM and DLS results. The reason might be caused by the increasing mass of the hydrophobic section after the grafting reaction in the diblock copolymer [24]. Whatever, all the results of SEM and DLS demonstrate the satisfactory self-assembly ability of **DOX-PNCHO-*b*-PNTEG** into micelles on the nanoscale, which is helpful for its future application in the field of medicine.

### In vitro pH-responsive release of DOX from micelles of DOX-PNCHO-b-PNTEG

**Figure 5.**
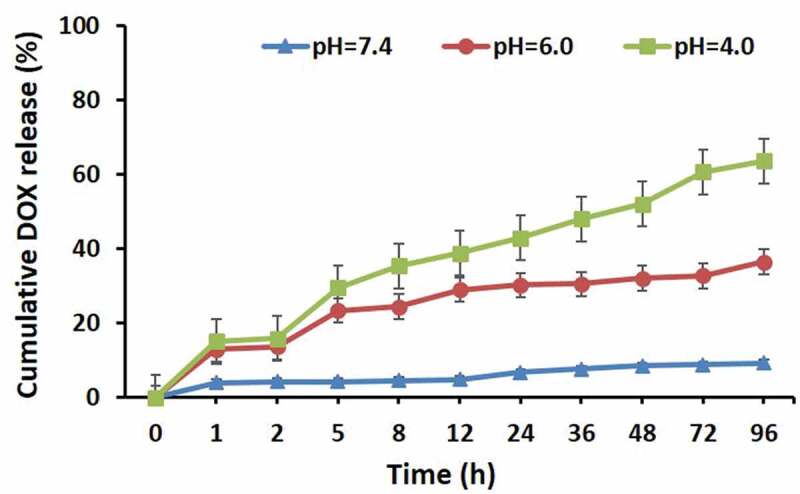
The *in vitro* cumulative release curves of DOX from micelles of **DOX-PNCHO-*b*-PNTEG** at different pH values

The pH-sensitive Schiff base imine bond (CH = N), formed by the reaction between primary amine (-NH_2_) and aldehyde (-CHO) groups, is well known for its decomposition under acidic conditions and moderate stability under alkaline environment [17]. It is reported that when the environmental pH is below 6.5, the Schiff base bond could be totally broken [18]. Thus, in the present study, the prepared conjugate of **DOX-PNCHO-*b*-PNTEG** with Schiff base linkage is anticipated to exhibit pH-responsive DOX release property. In simple terms, the loaded DOX could be released at acidic conditions owing to the broken action of CH = N bonds and be stopped when the environmental pH is neutral or alkaline. To verify this point, the *in vitro* DOX release experiments of micelles of **DOX-PNCHO-*b*-PNTEG** were conducted at pH 7.4, 6.0, and 4.0, respectively. And the release of DOX was monitored using the UV-vis spectroscopic. The characteristic absorption of DOX at a wavelength of 485 nm was measured at different time intervals, and the cumulative release amounts were calculated using the standard curve of DOX (Figure S20). Figure 5 provides the *in vitro* cumulative release curves of DOX from micelles of **DOX-PNCHO-*b*-PNTEG** at different pH values. As expected, the release behavior of DOX from micelles of **DOX-PNCHO-*b*-PNTEG** could be regulated by changing the environmental pH value. Specifically to say, under the pH value of 6.0, there is an obvious release of DOX, and after 96 h of dialysis treatment, the final cumulative release rate could reach 36.44%. When the pH value is decreased to 4.0, the release rate of DOX becomes faster. During the first 12 h of the dialysis process, the rapid release of DOX is observed, and the release rate is 38.81%; after 72 h, the release rate is increased to 60.60%;, and after 96 h, the final cumulative release rate reached to 63.52%. It is easy to find that the decrease of environmental pH could lead to the improvement of the final cumulative release amount of DOX. In contrast, when the pH value increases to 7.4, the final cumulative release rate is only 9.26% after 96 h of dialysis treatment. The above results indicate that the conjugated DOX molecules in **DOX-PNCHO-*b*-PNTEG** exhibit pH-responsive release behavior, and their release rate and amount could be regulated by varying the environmental pH value.

### In vitro toxicity to L-929 cells

**Figure 6.**
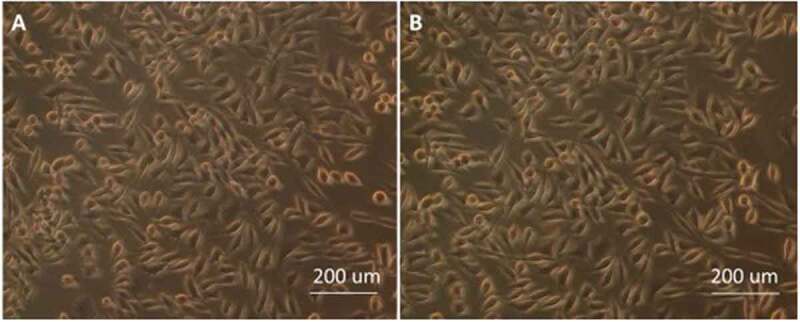
Photographs of adherent covered L929 cells after three days of culture. (a) Control group; (b) experimental group treated by **PNCHO-*b*-PNTEG** at 8.0 mg/mL

**Figure 7.**
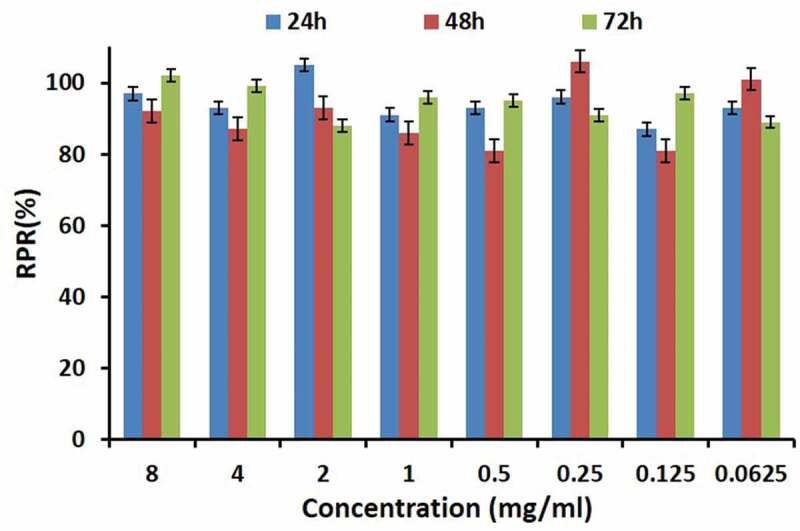
RPRs of L929 cells incubated with **PNCHO-*b*-PNTEG** at different concentrations

The biotoxicity evaluation of **PNCHO-*b*-PNTEG** was performed to further examine its application potential as a new polymeric carrier for drug delivery. It is *in vitro* cell toxicity that was firstly evaluated by using murine fibroblast cells, L-929 cells, as the tested object, and the typical CCK assay procedure [28] was conducted with the micelle concentrations of 8.0–0.0625 mg/mL. Figure 6 shows the visual cellular morphology of adherent covered L929 cells treated. After three days of culture, no inhibition of cell growth or cell malformation is observed in the experimental group treated by **PNCHO-*b*-PNTEG** at the maximum concentration of 8.0 mg/mL. There is no obvious difference in cellular morphology between the control and test groups (Figure 6). These results indicate that **PNCHO-*b*-PNTEG** has no obvious cell proliferation toxicity at the tested concentrations. The corresponding RPRs of L-929 cells (Figure 7) were further calculated at different concentrations of **PNCHO-*b*-PNTEG**. All the determined RPRs at different concentrations are beyond 80% during the 3 days of culture, and some of them are even beyond 100% and do not show significant difference compared to the control group. For example, when the L-929 cells were treated at the maximum concentration of 8.0 mg/mL, the calculated RPRs are 97%, 92%, and 102% for the first, second, and third days, respectively. There was no direct time dependence between the RPR value and contact time. For other concentrations, similar results are also obtained. According to the classification standard (Table S6), the cytotoxicity grade of **PNCHO-*b*-PNTEG** should be 0 or 1. In short, the above results demonstrate that **PNCHO-*b*-PNTEG** has excellent biocompatibility to L929 cells.

### In vivo acute embryonic developmental toxicity in zebrafish

**Figure 8.**
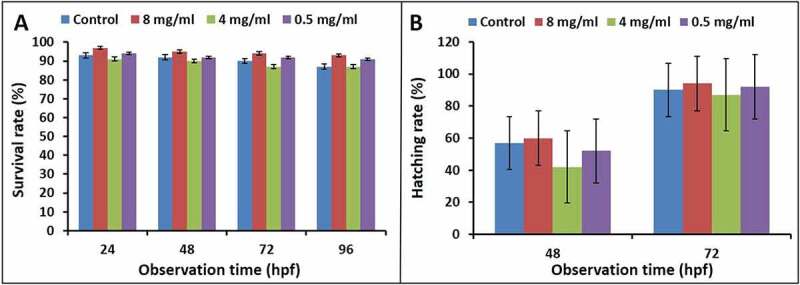
(a) Survival rates and (b) hatching rates of zebrafish embryos during the treatment with micelles of **PNCHO-*b*-PNTEG** at different concentrations

**Figure 9.**
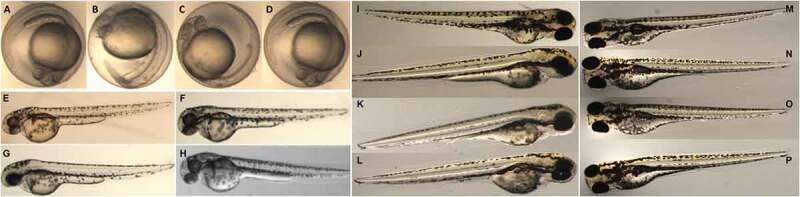
The photos of embryos of zebrafishes at 24 hpf treated by **PNCHO-*b*-PNTEG** with different concentrations of 0 (a, control), 8.0 (b), 4.0 (c), and 0.5 mg/mL (D). The photos of the zebrafish at 48 hpf treated by **PNCHO-*b*-PNTEG** with different concentrations of 0 (e, control), 8.0 (f), 4.0 (g), and 0.5 mg/ml (h). The photos of the zebrafish at 72 hpf treated by **PNCHO-*b*-PNTEG** with different concentrations of 0 (i, control), 8.0 (j), 4.0 (k), and 0.5 mg/ml (l). The photos of the zebrafish at 72 hpf treated by **PNCHO-*b*-PNTEG** with different concentrations of 0 (m, control), 8.0 (n), 4.0 (o), and 0.5 mg/ml (p)

The zebrafish embryo was adopted as a model animal to detect in vivo developmental toxicity of **PNCHO-*b*-PNTEG** to further evaluate the biosafety of **PNCHO-*b*-PNTEG** as a new polymeric carrier. The detection method and judge standard of each index were according to the reported literature [34]. In the present study, the survival and hatching rates of zebrafish embryos were determined at different concentrations (8, 4, and 0.5 mg/mL, respectively). As shown in Figure 8, during the whole experimental period up to 96 hpf, no obvious difference was observed between the control and experimental groups, indicating that the treatment of **PNCHO-*b*-PNTEG** at tested concentrations has no adverse effect on the survival and hatching rates of zebrafish embryos. Furthermore, the hatching process of zebrafish embryos and the development process of larvae were visually recorded by using a stereomicroscope, and the microscopic images at 24, 48, 72, and 96 hpf are shown in Figure 9. In both control and tested groups, zebrafish embryos exhibited good hatching and development features. For example, at 24 hpf (Figure 9(a-d)), all the zebrafish embryos exhibit normal characteristics at eye pigmentation and size, yolk pigmentation and shape, spinal development, and no tail adhesion or embryo agglutination occurred. At 96 hpf (Figure 9(m-p)), all the hatched larvae possess well-developed heads, notochords, caudal fins, eyes, tails, yolk sacs, pigmentation, etc. In a word, the above *in vivo* results confirm that **PNCHO-*b*-PNTEG** has no acute embryotoxicity in zebrafish.

### Cell death in live zebrafish

**Figure 10.**
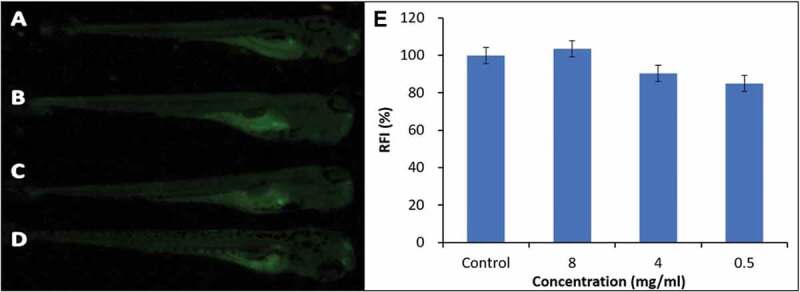
Digital photos of AO-dyed zebrafishes treated for 96 hpf using **PNCHO-*b*-PNTEG** at different concentrations. (a) control; (b) 8 mg/ml; (c) 4 mg/ml; (d) 0.5 mg/mL. (E) Fluorescence intensity of zebrafish embryo dyed by AO

As a nucleic acid selective fluorescent dye, AO has the ability to penetrate into apoptotic or necrotic cells and further combine with intracellular DNA or RNA molecules [35,36]. Bright green fluorescence could be observed for the combination of AO with DNA, while for the combination with RNA, orange-red or fire-red could be found. But, AO cannot enter into normal cells and exhibits non-toxicity to living cells [28]. Based on the above properties, herein, AO was adopted to stain the zebrafish larvae treated with **PNCHO-*b*-PNTEG** at different concentrations and images analyze were carried out employed Image J for quantifying the fluorescence intensity, and the toxicity of this polymeric carrier was thus evaluated from the level of cell death. In the control group (Figure 10(a)), weak green fluorescence is significantly observed at the yolk sac of zebrafish larva after 96 hpf, which is attributed to the voluntary and orderly apoptosis controlled by genes [35,36]. Unlike the cell necrosis under pathological conditions, cell apoptosis, involving the activation, expression, and regulation of a series of genes, is an active death process to better adapt to the living environment [28]. For the experimental groups, weak green fluorescence is also observed at the yolk sac of zebrafish larvae after 96 hpf (Figure 10(b-d)), and there is no significant difference in fluorescence intensity between the control and treated groups (Quantitative analysis of the fluorescence intensity see Figure 10(e)). These results indicate that **PNCHO-*b*-PNTEG** cannot lead to abnormal cell death during the development of zebrafish embryos, which further demonstrates the excellent biocompatibility of the new diblock copolymer.

## Conclusion

A novel polynorbornene-based amphiphilic diblock copolymer **PNCHO-*b*-PNTEG** with a well-defined structure was successfully and rapidly synthesized *via* the ROMP technique in the presence of the Grubbs’ 3rd generation catalyst as an initiator. The formation, structure, molecular weight, and the targeted polymerization degrees for the two blocks of **PNCHO-*b*-PNTEG** were fully demonstrated by various analytical methods. This amphiphilic diblock copolymer could self-assemble into near-spherical micelles with the particle size of 76 ± 10 nm in aqueous solution. More importantly, **PNCHO-*b*-PNTEG** could be used as a polymeric carrier to conjugate efficiently amino-containing molecules with the grafting rate of 100% *via* the Schiff base reaction of its side-chain aldehyde groups with the amino groups of loaded molecules. The formed conjugate with DOX, **DOX-PNCHO-*b*-PNTEG** also possesses amphiphilicity and self-assembles in water into micelles with near-spherical morphology and the particle size of 81 ± 10 nm. The conjugated DOX molecules exhibit pH-sensitive release behavior, and their release rate and amount could be well regulated by varying the environmental pH value. Furthermore, the excellent biological safety of **PNCHO-*b*-PNTEG** is adequately confirmed by the results from both *in vitro* toxicity evaluation to L-929 cells and *in vivo* evaluation of embryotoxicity and cell death in zebrafish. Consequently, copolymer **PNCHO-*b*-PNTEG** is expected to find potential applications in the field of medicine as a promising polymeric carrier to conjugate amino-containing drug molecules *via* Schiﬀ base reaction.

## Supporting information

Information is available regarding the preparation and characterization of **PNCHO-*b*-PNTEG, DOX-PNCHO-*b*-PNTEG** and their self-assembly. The materials are available via the Internet at http://www.springer.com/13233.
